# Autophagy-dependent and -independent modulation of oxidative and organellar stress in the diabetic heart by glucose-lowering drugs

**DOI:** 10.1186/s12933-020-01041-4

**Published:** 2020-05-13

**Authors:** Milton Packer

**Affiliations:** 1 Baylor Heart and Vascular Institute, Baylor University Medical Center, 621 N. Hall Street, Dallas, TX 75226 USA; 2 Imperial College, London, UK

**Keywords:** Autophagy, SGLT2 inhibitors, Metformin, Sirtuin-1, Uric acid

## Abstract

Autophagy is a lysosome-dependent intracellular degradative pathway, which mediates the cellular adaptation to nutrient and oxygen depletion as well as to oxidative and endoplasmic reticulum stress. The molecular mechanisms that stimulate autophagy include the activation of energy deprivation sensors, sirtuin-1 (SIRT1) and adenosine monophosphate-activated protein kinase (AMPK). These enzymes not only promote organellar integrity directly, but they also enhance autophagic flux, which leads to the removal of dysfunctional mitochondria and peroxisomes. Type 2 diabetes is characterized by suppression of SIRT1 and AMPK signaling as well as an impairment of autophagy; these derangements contribute to an increase in oxidative stress and the development of cardiomyopathy. Antihyperglycemic drugs that signal through insulin may further suppress autophagy and worsen heart failure. In contrast, metformin and SGLT2 inhibitors activate SIRT1 and/or AMPK and promote autophagic flux to varying degrees in cardiomyocytes, which may explain their benefits in experimental cardiomyopathy. However, metformin and SGLT2 inhibitors differ meaningfully in the molecular mechanisms that underlie their effects on the heart. Whereas metformin primarily acts as an agonist of AMPK, SGLT2 inhibitors induce a fasting-like state that is accompanied by ketogenesis, a biomarker of enhanced SIRT1 signaling. Preferential SIRT1 activation may also explain the ability of SGLT2 inhibitors to stimulate erythropoiesis and reduce uric acid (a biomarker of oxidative stress)—effects that are not seen with metformin. Changes in both hematocrit and serum urate are the most important predictors of the ability of SGLT2 inhibitors to reduce the risk of cardiovascular death and hospitalization for heart failure in large-scale trials. Metformin and SGLT2 inhibitors may also differ in their ability to mitigate diabetes-related increases in intracellular sodium concentration and its adverse effects on mitochondrial functional integrity. Differences in the actions of SGLT2 inhibitors and metformin may reflect the distinctive molecular pathways that explain differences in the cardioprotective effects of these drugs.

## Background

Autophagy is an evolutionarily-conserved intracellular degradative pathway, which mediates the cellular adaptation to stressful conditions. Autophagy involves the enclosure of unwanted cytosolic constituents by an autophagosome membrane, and the contents of these vesicles are destroyed when they fuse with lysosomes [1]. When stimulated nonselectively, autophagy recycles cellular components to generate ATP to support cells that are energy starved. Yet, autophagy can also be activated selectively in order to rid cells of accumulated debris, excessive stores of glucose and lipids, unfolded proteins, and dysfunctional or damaged organelles, which are seminal to the pathogenesis of disease [1, 2].

## Triggers of and molecular pathways leading to autophagy

The primordial stimulus to autophagy is energy starvation—specifically, nutrient and oxygen deprivation. However, autophagic flux is also activated in response to a broad range of cellular stresses, including oxidative and endoplasmic reticulum stress. The most important sources of oxidative stress are dysfunctional mitochondria and peroxisomes, the two major oxygen-consuming constituents in the cell [3]. Endoplasmic reticulum stress is caused by the accumulation of misfolded proteins, glycation endproducts or fatty acid intermediates [4]. Regardless of the triggering event, autophagy is part of a wide-ranging transcriptional and metabolic shift that promotes cellular and organismal survival by prioritizing maintenance over growth [5]. Autophagy underlies the effect of starvation to prolong life in a broad range of animal species; tissue-specific overexpression of single autophagy genes is sufficient to extend lifespan [6]. Conversely, impairment of autophagy has been implicated in the pathogenesis of many human illnesses, including metabolic, cardiovascular, neurodegenerative and autoimmune diseases, and cancer [1, 2].

### Nutrient and oxygen deprivation signaling promotes autophagic flux

**Fig. 1:**
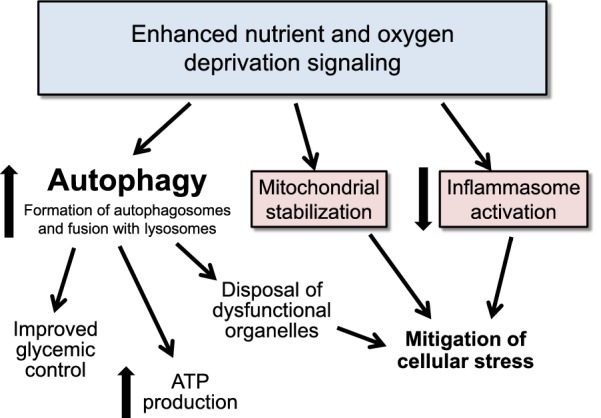
Effect of enhanced nutrient and oxygen deprivation signaling on autophagic flux, mitochondrial homeostasis and inflammasome activation. ATP: adenosine triphosphate

The molecular mechanisms that can activate autophagy are complex (Fig. 1). Nutrient deprivation leads to increased expression and activity of master regulator enzymes, which include sirtuin-1 (SIRT1) and adenosine monophosphate-activated protein kinase (AMPK) [7]. SIRT1 responds to levels of nicotinamide adenine dinucleotide and serves as a redox rheostat; its activation serves to support blood levels of glucose [8, 9]. AMPK is sensitive to the balance between ATP and ADP or AMP in the cytosol; its activation leads to the breakdown of energy stores, thereby promoting the generation of ATP [10]. Oxygen deprivation leads to increased expression and activity of hypoxia inducible factors (HIF-1α and HIF-2α), which promote the delivery and reduce the utilization of oxygen [11].

SIRT1, AMPK, HIF-1α and HIF-2α are master regulators of hundreds of genes and proteins that play a critical role in maintaining cellular homeostasis, and they can augment autophagy in cardiomyocytes and in diabetic hearts under stress [12–15]. The interplay of HIF-1α with beclin 1 promotes autophagosome formation [16], and phosphorylation of AMPK causes dissociation of the beclin 1-Bcl2 complex [12] and enhances the maturation of autophagosomes and their fusion with lysosomes [17]. In contrast, SIRT1 and HIF-2α act primarily to enhance selective autophagy, i.e., SIRT1 promotes the clearance of damaged mitochondria [18], whereas HIF-2α stimulates the degradation of dysfunctional peroxisomes [19]. Consistent with their intertwined functions, SIRT1 and HIF-2α augment and reinforce each other [20, 21].

### Nutrient and oxygen deprivation signaling can mitigate oxidative stress and inflammation through mechanisms that are not autophagy-dependent

Nutrient and oxygen deprivation signaling can influence oxidative stress and inflammatory pathways in ways that may be independent of their effects to promote autophagy (Fig. 1). Both SIRT1 and AMPK act directly to maintain mitochondrial network homeostasis [22–24] and preserve peroxisome functionality [24, 25], and they enhance the activity of antioxidant enzymes [26]. Additionally, both SIRT1 and AMPK interact with a key subunit of NFκB to inhibit its actions, thereby attenuating activation of the NLRP3 inflammasome and muting inflammation-mediated cellular injury [27, 28]. HIF-2α shifts the cellular milieu towards an antioxidant state [29], and HIF-2α upregulation is accompanied by an anti-inflammatory macrophage polarization phenotype [30], potentially explaining why HIF-2α acts to mute the inflammatory response that underlies insulin resistance in obesity [31].

Therefore, acting through both autophagy-dependent or -independent mechanisms, the interplay of SIRT1 and HIF-2α plays a major role in ameliorating oxidative stress in the heart. Activation of SIRT1 decreases the production of reactive oxygen species [32, 33], whereas genetic or pharmacological suppression of SIRT1 markedly augments oxidative stress [34, 35]. Similarly, degradation or inhibition of HIF-2α acts to undermine antioxidant mechanisms [29, 36, 37], whereas activation of HIF-2α by cobalt chloride reduces oxidative stress in cardiac and vascular tissues [38–40]. If the levels of SIRT1 and HIF-2α decline, the resulting increase in oxygen free radicals acts to reactivate SIRT1 and HIF-2α signaling [41, 42], thereby limiting oxygen-mediated cellular stress.

## Suppression of autophagic flux and nutrient deprivation sensor signaling in type 2 diabetes

Type 2 diabetes is characterized by hyperglycemia and hyperinsulinemia and is typically accompanied by the intracellular accumulation of glycogen and lipids. The accumulation of glycation and fatty acid intermediates undermines mitochondrial and peroxisomal stability, leading to the production of reactive oxygen species and oxidative stress [43]. The overabundance of nutrients also promotes the formation of unfolded proteins and potentially toxic lipid pools, which cause endoplasmic reticular stress [44, 45]. When these changes occur in the heart, the result is cardiomyocyte dysfunction and demise.

**Fig. 2:**
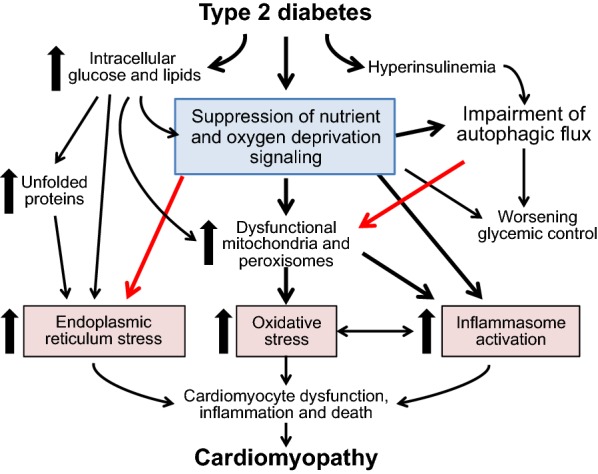
Derangements in energy deprivation signaling in type 2 diabetes and its implications for the development of cardiomyopathy

Although cells might be able to mitigate these metabolic, oxidative and endoplasmic reticulum stresses by stimulating autophagic flux, the stimulation of and capacity for autophagy is markedly impaired in states of energy surplus (Fig. 2) [46, 47]. Type 2 diabetes is accompanied by a decrease in the activation of SIRT1 and AMPK and by a striking suppression of autophagy [48–50]; these changes have been implicated in the myocardial injury and cardiomyopathy in type 2 diabetes [49–51]. Activation of SIRT1 alleviates oxidative stress, promotes autophagic flux, and prevents cardiomyocyte dysfunction and demise in diabetic hearts [13, 52–54]. Similarly, a high-fat diet acts to suppress (whereas glucose deprivation activates) HIF-2α [55–57], whereas upregulation of HIF-2α reduces oxidative stress and promotes autophagy in the heart [38, 39]. Thus, changes in nutrient and oxygen deprivation signaling can influence organellar stability, oxidative stress and inflammasome activation and modulate cellular dysfunction in diabetic hearts by mechanisms that are autophagy-dependent and -independent.

Interestingly, the energy surplus in type 2 diabetes may not only lead to the suppression of low-energy sensors, but changes in SIRT1, AMPK, HIF-1α and HIF-2α signaling may also contribute to glucose intolerance. Stimulation of SIRT1 and AMPK improves glycemic control, glucose transporter expression and insulin sensitivity [58–61], and intermittent hypoxia improves glycemia by causing upregulation of both AMPK and HIF-1α [61, 62]. Activation of HIF-1α enhances glycolysis, whereas HIF-2α suppresses gluconeogenesis; [63, 64] additionally, HIF-2α enhances insulin sensitivity and inhibits the actions of glucagon [64, 65]. The coordinated effects of hypoxia inducible factor signaling act to lower blood glucose, while simultaneously mediating the adaptation of cells to hypoglycemia [66]. Interestingly, the benefits of enhanced SIRT1/AMPK/HIF signaling on glucose homeostasis are likely to be mediated (at least in part) through enhanced autophagic flux, which plays a critically important role in promoting normal glucose utilization [67].

## Effect of antihyperglycemic drugs on autophagic flux, nutrient deprivation signaling and cellular stress

Theoretically, any antihyperglycemic drug might increase the activity of low-energy sensors and promote autophagy simply by lowering blood glucose; however, the magnitude of the effect may be modest and be offset by other actions. Incretins and thiazolidinediones have been reported to enhance autophagy in experimental models [68–70], but they potentiate the release and/or response to insulin, which acts to suppress autophagy [71]. These effects may help to explain why enhanced insulin signaling adversely affects the course of heart failure [72]. In addition, dipeptidyl peptidase 4 inhibitors potentiate the actions of stromal cell-derived factor 1, which signals through its receptor CXCR4 to depress autophagic flux [73, 74].

**Fig. 3:**
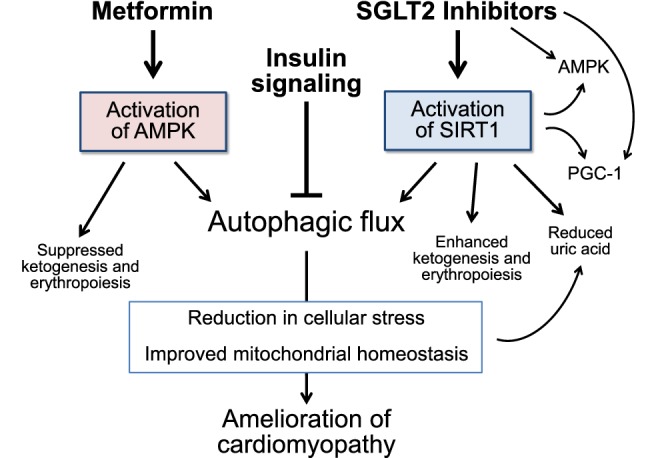
Mechanisms underlying the effects of glucose-lowering drugs to influence the development of cardiomyopathy. The possibility that glycosuria produced by SGLT2 inhibitors can promote renal urate excretion is not shown. AMPK: adenosine monophosphate-activated protein kinase; PGC-1α: peroxisome proliferator-activated receptor-γ coactivator-1α; SIRT1: sirtuin-1

Two glucose-lowering drugs—metformin and SGLT2 inhibitors—promote nutrient deprivation signaling and autophagic flux without enhancing insulin signaling (Fig. 3).

### Effects of metformin on nutrient and oxygen deprivation signaling and autophagic flux in diabetic and nondiabetic hearts under stress

Metformin promotes autophagy in hearts under stress, and this action may contribute to the effect of the drug to ameliorate cardiomyocyte dysfunction and the evolution of experimental cardiomyopathy, in the presence or absence of diabetes [75–78]. The effect of metformin to promote autophagy is primarily related to its ability to act as an agonist of AMPK [76, 79], but signaling through AMPK is capable of ameliorating oxidative stress and cardiac inflammation in ways that are independent of changes in autophagic flux [80–83]. Additionally, metformin may produce cardioprotective effects that are independent of AMPK [84], potentially by suppressing the activity of the Akt/mTOR pathway [85].

Although metformin has been postulated to interact with SIRT1 [86] several lines of evidence suggest that its capacity to promote SIRT1 signaling is modest and is not likely to mediate the cardioprotective effects of the drug. As expected from an AMPK agonist, metformin suppresses gluconeogenesis [87], but drugs that act through SIRT1 stimulate gluconeogenesis [10]. SIRT1 activation is also expected to promote erythropoiesis (since SIRT1 stimulates HIF-2α [20]); yet, metformin decreases the hematocrit [88], presumably because signaling through AMPK acts to suppress HIF-2α [89].

### Effects of SGLT2 inhibitors on nutrient and oxygen deprivation signaling, autophagic flux and organellar dysfunction in cardiomyocytes

SGLT2 inhibitors cause loss of calories in the urine, and as a result of the induction of a starvation-like state [90], SGLT2 inhibitors stimulate the activity of SIRT1 [91–94] the principal sensor of glucose depletion. SGLT2 inhibitors also upregulate another nutrient deprivation sensor, peroxisome proliferator-activated receptor-γ coactivator-1α (PGC-1α) [95, 96], the downstream target of SIRT1 and a master regulator of mitochondrial biogenesis (Fig. 3). In addition, certain SGLT2 inhibitors (e.g., canagliflozin) directly activate AMPK; [97–99] empagliflozin and dapagliflozin may also promote AMPK activity, although not necessarily by a direct action or to a meaningful degree [92, 97–102]. The effects of SGLT2 inhibitors on hypoxia inducible factors in the heart have not been evaluated to date.

The action of SGLT2 inhibitors to stimulate SIRT1 (alone or in concert with other nutrient deprivation sensors) may explain the ability of these drugs to maintain mitochondrial membrane potential, preserve mitochondrial structure, restore the capacity of mitochondria to generate ATP, and mitigate mitochondrial fragmentation and DNA injury [101–104]. These benefits may be achieved by a direct salutary effect of SIRT1/AMPK/PGC-1α signaling on existing mitochondria; through autophagic clearance of injured mitochondria; and by promoting the biogenesis of healthy mitochondria (Fig. 3) [18, 22–25, 105, 106]. SGLT2 inhibitors have been shown to promote autophagic flux in diabetic hearts, thereby muting inflammation [100]. The autophagy-dependent and -independent action of SGLT2 inhibitors to maintain organellar health likely underlies their ability to ameliorate the course of experimental diabetic and nondiabetic cardiomyopathy [107–109].

Interestingly, intracellular sodium concentration is increased in cardiomyocytes derived from diabetic hearts [110, 111]; this perturbation may compromise the capacity of mitochondria to generate ATP and reduce the generation of reactive oxygen species [112, 113]. It is therefore noteworthy that SIRT1/AMPK signaling modulates the activity of transporters so as to promote sodium efflux out of cells [49, 114–116]; the resulting decrease in intracellular sodium concentrations improves mitochondrial function and antioxidant defense mechanisms, thereby preventing cell death [112]. Interestingly, SGLT2 inhibitors have been shown to decrease intracellular sodium concentration in cardiomyocytes [117]. Although this finding has been attributed to an effect on sodium-hydrogen exchange in the heart, an effect of SGLT2 inhibitors on the exchanger has yet to be demonstrated. Instead, the effect of these drugs on cytosolic sodium may possibly be the result of AMPK/SIRT1 signaling.

It is important to recognize that the effects of SGLT2 inhibitors to promote SIRT1/AMPK signaling are not cardiac specific. The loss of calorie in the urine triggers a system-wide starvation prosurvival transcriptional paradigm in a broad range of tissues [91]. Specifically, glycosuria stimulates SIRT1 in the liver and promotes hepatic gluconeogenesis, even though SGLT2 is not expressed in hepatic tissues [92]. SGLT2 inhibitors ameliorate the structural and functional derangements in the heart, liver, kidney, adipose tissue and skeletal muscle that are seen in states of energy overabundance [92, 118, 119], even though there are no measurable levels of the target protein in most of these tissues.

### Distinctions between metformin and SGLT2 inhibitors with respect to energy deprivation signaling and cardioprotection

There is compelling evidence from large-scale trials that SGLT2 inhibitors reduce the risk of cardiovascular death and hospitalization for heart failure in patients with and without diabetes [120, 121]. In contrast, there is uncertainty whether metformin exerts such benefits in the clinical setting. Metformin has been associated with a reduction in heart failure events in some (but not all) epidemiological studies [122–125]; however, in these reports, metformin was compared with antihyperglycemic drugs that can increase the risk of heart failure. Furthermore, in these studies, it seems likely that metformin was preferentially prescribed to patients at low risk of heart failure [126], since physicians have worried that the drug may trigger lactic acidosis. Given the observational nature of these analyses and the lack of evidence from randomized controlled trials, the true effect of metformin on the development of heart failure in patients with type 2 diabetes remains unclear [127].

However, metformin and SGLT2 inhibitors differ with respect to their actions to promote nutrient and oxygen deprivation signaling (Fig. 3). Metformin exerts its effects primarily through the activation of AMPK; in contrast, several lines of evidence suggest that SGLT2 inhibitors exert their effects principally through SIRT1 and its downstream effectors, and not AMPK [95–99]. Due to the loss of calories in the urine, SGLT2 inhibitors recapitulate a starvation-like state, which signals more through SIRT than AMPK [128–130], since SIRT1 (and not AMPK) mediates the effects of caloric restriction to prolong survival [131]. Additionally, both fasting and SGLT2 inhibition are accompanied by hyperketonemia, and there is a close association between ketogenesis and the activation of SIRT1 [132–134] Ketogenesis depends on gluconeogenesis, which is stimulated by SIRT1 [1, 10] but inhibited by AMPK and metformin [135, 136]. The other major pathway leading to the formation of ketone bodies—fatty acid oxidation—also requires SIRT1 [137–139]. Finally, pretreatment with metformin does not attenuate the ability of empagliflozin (which does not directly activate AMPK [97, 98]) to reduce the risk of heart failure hospitalizations [140]. Therefore, in contradistinction to metformin, it appears that SGLT2 inhibitors preferentially activate SIRT1, rather than AMPK.

Differences in the pattern of nutrient deprivation signaling with metformin and SGLT2 inhibitors may also lead to different effects on intracellular sodium. As noted earlier, SGLT2 inhibitors reduce levels of cytosolic sodium in cardiomyocytes, an effect that may yield direct benefits on mitochondrial capacity and stability [112, 117]. In contrast, metformin does not ameliorate the heightened intracellular sodium concentrations seen in diabetic cardiomyocytes [110, 111, 141].

### SIRT1 signaling may explain the results of statistical mediation analyses of the heart failure benefit seen in large-scale clinical trials

The likely role of SIRT1 in mediating the effects of SGLT2 inhibitors is noteworthy, since SIRT1 (but not AMPK) can stimulate HIF-2α [20, 21], the primary transactivator of the gene for erythropoietin synthesis [142]. Interestingly, SGLT2 inhibitors have been strongly linked to the enhanced production of erythropoietin and to an increase in red blood cell mass in clinical trials [121, 143–145]. More importantly, activation of HIF-2α can be expected to exert its own effects to promote autophagy and mute cellular stress and inflammation [19, 29–31]. In contrast, as a result of AMPK agonism, metformin suppresses the activity of HIF-2α [89], and thus, the drug decreases the hematocrit [88]. The potential differences in HIF-2α signaling between SGLT2 inhibitors and metformin may be clinically relevant, since (in statistical mediation analyses) the erythrocytosis produced by SGLT2 inhibitors is the most powerful predictor of the ability of these drugs to reduce the risk of serious heart failure events in large-scale clinical trials [144, 145].

Interestingly, in the mediation analyses of large-scale cardiovascular outcomes trials, the effect of SGLT2 inhibitors to decrease serum uric acid is also a major independent predictor of the drug-related reduction in serious heart failure events [144, 145]. Previous work attributed the urate-lowering effects of SGLT2 inhibitors to an effect of these drugs to simultaneously inhibit glucose and uric acid reabsorption in the proximal renal tubule [146], since glycosuria may directly enhance fractional excretion of uric acid [147]. However, urate is also a biomarker of oxidative stress in the stressed myocardium [148–150], i.e., the increase in reactive oxygen species in patients with diabetes leads to activation of xanthine oxidase, the enzyme that catalyzes the synthesis of uric acid [151]. Interestingly, the depletion of nicotinamide adenine dinucleotide (NAD+) in diabetes not only causes upregulation of xanthine oxidase but also downregulation of SIRT1 [152, 153]. There is an inverse relationship between the activities of SIRT1 and xanthine oxidase. Upregulation of xanthine oxidase suppresses SIRT1 [154] and inhibition of xanthine oxidase activates SIRT1 [155]; thus, serum levels of uric acid are inversely related to the activity of SIRT1 in states of energy overabundance [156]. Therefore, by enhancing SIRT1-mediated suppression of oxidative stress or by a direct consequence of SIRT1 activation [157–159], SGLT2 inhibitors may suppress the activity of xanthine oxidase and reduce serum uric acid [145, 160]. Thus, activation of SIRT1 may explain the observed statistical link between the urate-lowering and cardioprotective effects of SGLT2 inhibitors. In contrast, metformin (which does not enhance signaling through SIRT1) increases serum uric acid [161].

## Conclusions

Heart failure is the most common and serious cardiovascular complication of type 2 diabetes, possibly because diabetes increases oxidative and endoplasmic reticulum stress in cardiomyocytes, with its attendant risks of cellular dysfunction and demise. The increase in cellular stress in the diabetic heart is related to suppression of nutrient deprivation signaling, which normally acts to maintain organellar function and promote the removal of dysfunctional mitochrondria and peroxisomes through the lysosome-dependent housekeeping process of autophagy. The downregulation of SIRT1 and AMPK has been shown to cause cardiomyopathy in experimental models of diabetes, whose features are characterized by oxidative stress and organellar dysfunction.

Both metformin and SGLT2 inhibitors activate SIRT1 and AMPK, which may explain their effect to alleviate cellular stress and ameliorate the course of experimental cardiomyopathy, benefits that are likely mediated through their actions to restore mitochondrial function, both directly and indirectly, through their actions to promote autophagy. However, the evidence supporting a heart failure benefit is substantially more compelling with SGLT2 inhibitors than with metformin. Furthermore, SGLT2 inhibitors may have important mechanistic advantages over metformin in producing cardioprotection. Specifically, they may preferentially enhance SIRT1 and HIF-2α (as reflected by ketogenesis and erythrocytosis), alleviate sources of oxidative stress (as reflected by serum uric acid levels), and reduce intracellular sodium concentration in cardiomyocytes—effects that are not seen with metformin. Therefore, differences in their action on nutrient deprivation pathways may underlie differences between metformin and SGLT2 inhibitors in their ability to reduce heart failure events in the clinical setting.

## Data Availability

Not applicable.

## Abbreviations

ADP: Adenosine diphosphate
Akt: Protein kinase B
AMP: Adenosine monophosphate
AMPK: Adenosine monophosphate-activated protein kinase
ATP: Adenosine triphosphate
CXCR4: C-X-C chemokine receptor type 4
HIF: Hypoxia inducible factor
HIF-1α: Hypoxia inducible factor isoform 1-alpha
HIF-2α: Hypoxia inducible factor isoform 2-alpha
mTOR: Mammalian target of rapamycin
NFκB: Nuclear factor kappa-light-chain-enhancer of activated B cells
NLRP3: NOD-, LRR- and pyrin domain-containing protein 3
PGC-1α: Peroxisome proliferator-activated receptor-γ coactivator-1α
SGLT2: Sodium-glucose transporter 2
SIRT1: Sirtuin-1
